# HCMV-secreted glycoprotein gpUL4 inhibits TRAIL-mediated apoptosis and NK cell activation

**DOI:** 10.1073/pnas.2309077120

**Published:** 2023-11-27

**Authors:** Virginia-Maria Vlachava, Sepehr Seirafian, Ceri A. Fielding, Simon Kollnberger, Rebecca J. Aicheler, Joseph Hughes, Alexander Baker, Michael P. Weekes, Simone Forbes, Gavin W. G. Wilkinson, Eddie C. Y. Wang, Richard J. Stanton

**Affiliations:** ^a^Infection and Immunity, School of Medicine, Cardiff University, Cardiff CF14 4XN, United Kingdom; ^b^Department of Biomedical Sciences, Cardiff School of Sport and Health Sciences, Cardiff Metropolitan University, Cardiff CF5 2YB, United Kingdom; ^c^Centre for Virus Research, School of Infection & Immunity, Glasgow University, Glasgow G61 1QH, United Kingdom; ^d^Cambridge Institute for Medical Research, Department of Medicine, University of Cambridge, Cambridge CB2 0XY, United Kingdom

**Keywords:** HCMV, NK Cells, TRAIL

## Abstract

Human cytomegalovirus (HCMV) is a paradigm of pathogen immune evasion and sustains lifelong persistent infection in the face of exceptionally powerful host immune responses through the concerted action of multiple immune-evasins. These reduce NK cell activation by inhibiting ligands for activating receptors, expressing ligands for inhibitory receptors, or inhibiting synapse formation. However, these functions only inhibit direct interactions with the infected cell. To determine whether the virus also expresses soluble factors that could modulate NK function at a distance, we systematically screened all 170 HCMV canonical protein-coding genes. This revealed that UL4 encodes a secreted and heavily glycosylated protein (gpUL4) that is expressed with late-phase kinetics and is capable of inhibiting NK cell degranulation. Analyses of gpUL4 binding partners by mass spectrometry identified an interaction with TRAIL. gpUL4 bound TRAIL with picomolar affinity and prevented TRAIL from binding its receptor, thus acting as a TRAIL decoy receptor. TRAIL is found in both soluble and membrane-bound forms, with expression of the membrane-bound form strongly up-regulated on NK cells in response to interferon. gpUL4 inhibited apoptosis induced by soluble TRAIL, while also binding to the NK cell surface in a TRAIL-dependent manner, where it blocked NK cell degranulation and cytokine secretion. gpUL4 therefore acts as an immune-evasin by inhibiting both soluble and membrane-bound TRAIL and is a viral-encoded TRAIL decoy receptor. Interestingly, gpUL4 could also suppress NK responses to heterologous viruses, suggesting that it may act as a systemic virally encoded immunosuppressive agent.

## Significance

NK cells are critical to control HCMV, and the virus encodes over twelve genes that inhibit their activity. Investigating these immune-evasins has not only revealed details of virus pathogenesis but also mechanisms of immune cell function. Previously documented HCMV NK-evasins only inhibit the ability of NK cells to recognise the infected cell itself. In contrast, gpUL4 can operate in the area surrounding the infected cell, inhibiting both NK cell activity and bystander cell death. Here, it can promote an immunosuppressive microenvironment and influence the NK response to other pathogens, such as SARS-CoV-2. In addition, the ability of gpUL4 to inhibit NK cell degranulation through TRAIL supports a role for TRAIL in NK cell activation.

Human cytomegalovirus (HCMV) is a clinically important human herpesvirus that produces a lifelong persistent infection. HCMV infects a broad range of cell types in vivo, consistent with its association with a wide variety of end-organ diseases. Myeloid cells act as a vehicle for systemic spread and lifelong virus reactivation; HCMV infects myeloid progenitors, is carried by monocytes then ultimately reactivates following differentiation into macrophages, dendritic or Langerhans cells (1). Continuous reactivation in these professional antigen-presenting cells, combined with productive infection in other cell types, provides a lifelong chronic immune stimulus. Seroconversion is accompanied by profound changes to the immune cell repertoire. An inflationary cytotoxic T cell response can result in up to 30% of peripheral CD8+ T cells being HCMV specific (2). Moreover, clonal expansions of atypical “adaptive” NK cell subsets capable of antibody-dependent cellular cytotoxicity (ADCC) may contribute to both innate and adaptive control of infections (3, 4). To counter host immunity, HCMV encodes a broad repertoire of immune-evasion functions, and many have been implicated in both the induction and sculpting of these atypical adaptive NK and T cell expansions (5).

The HCMV genome is the largest of any characterised human virus, containing 170 canonical protein-coding genes of which only 41 to 45 are essential for replication in vitro (6, 7). The majority of its protein-coding capacity therefore consists of accessory gene functions that have been selected to promote survival in vivo. The characterisation of HCMV accessory genes has informed our understanding both of virus pathogenesis and the underlying function of the human immune system (5, 8). HCMV has an extraordinary capacity to interfere with host immune defences; the virus encodes an inhibitor of immune synapse formation (9), ≥4 genes that suppress MHC-I antigen presentation, ≥12 functions that interfere with NK cell receptor recognition through their impact on either inhibitory (10–12) or activating ligands (13–24), plus a gene family whose 10 members modulate cell surface immune ligands (17). These viral immune-evasins act in concert to suppress recognition and responses to the infected cell. While HCMV is a paradigm of viral immune evasion, it is important to recognise that the virus also has the potential to be immunosuppressive. In this context, HCMV-infected cells are known to secrete both a functional IL-10 mimic (cmvIL-10) (25, 26) and a decoy receptor for RANTES (pUL22A) (27, 28).

Despite the identification of an impressive arsenal of immune-modulators, the functions of many accessory genes in the HCMV genome are not known. To facilitate the identification and characterization of novel HCMV gene functions, we cloned all 170 canonical genes into an adenovirus (Ad) vector (29). During the characterization of this expression library, we observed that the Ad UL4 construct was remarkable in encoding a protein that was secreted from cells in high abundance. UL4 is one of 15 members of the RL11 gene family (30). Most of this family are predicted to encode cell surface glycoproteins with an immunoglobulin fold, although UL4 is conspicuous in lacking a transmembrane domain. We therefore sought first to determine whether the UL4 gene product was also secreted from HCMV-infected cells and, if so, to investigate its potential role as an immunomodulatory factor capable of systemically impacting host immunity.

## Results

### UL4 Encodes a Secreted Glycoprotein.

**Fig. 1.**
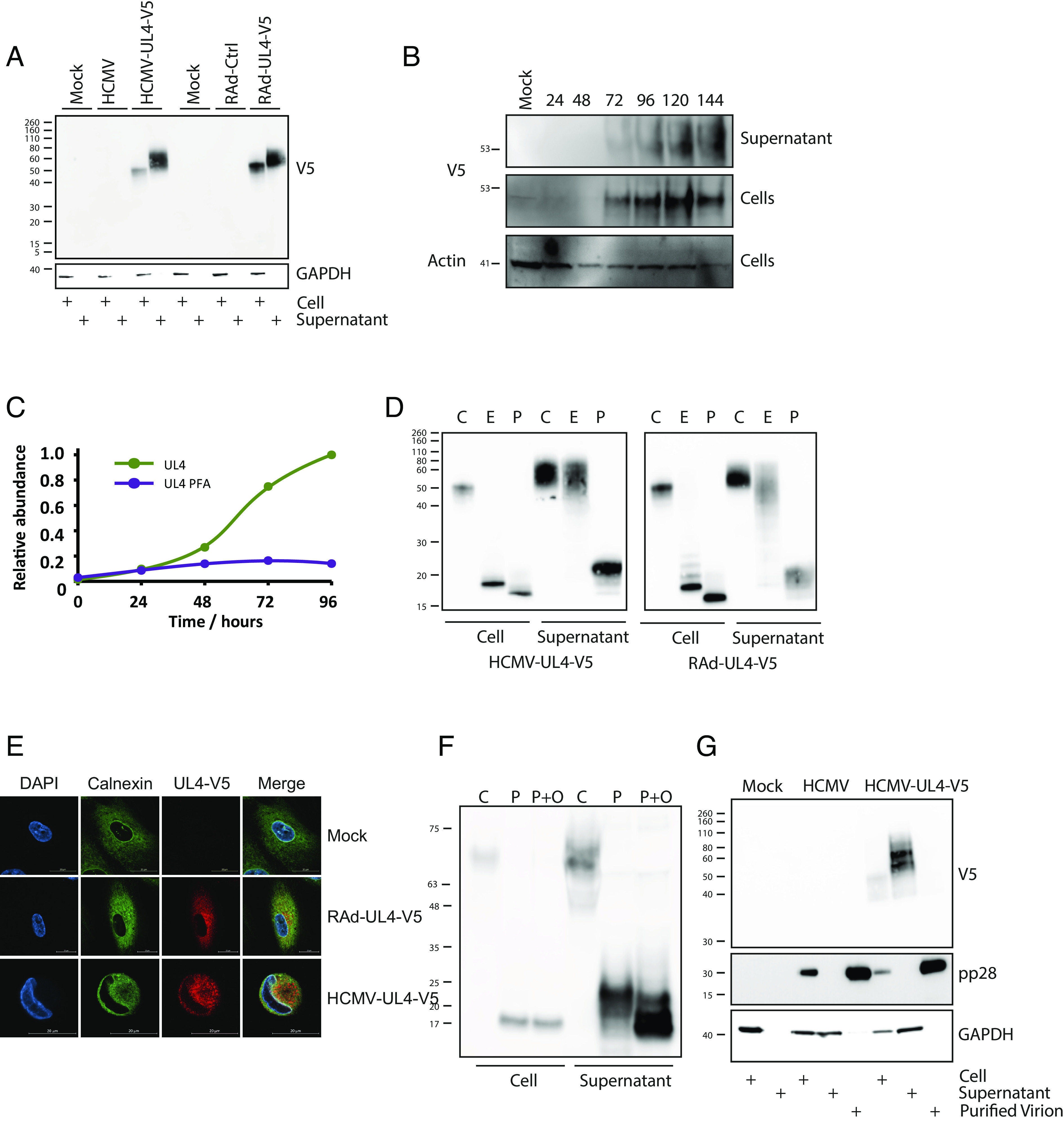
UL4 is a highly glycosylated soluble protein expressed during HCMV infection. HFFF-hTert were Mock infected, or infected with HCMV, or HCMV containing a C-terminal V5 tagged UL4 (HCMV-UL4-V5) for 96 h, or the indicated timepoints, at MOI = 5. Alternatively, HFFF-hCAR were infected with RAd vectors for 48 h. UL4 expression was detected using a V5 antibody in all cases. (*A*) Whole cell lysates (“Cell”) or supernatants were analysed by SDS-PAGE followed by western blot and stained as indicated. (*B*) Whole cell lysates or supernatants were collected daily from 24 to 144 h postinfection and analysed by SDS-PAGE and western blot for the indicated proteins. (*C*) Expression of gpUL4 as determined by proteomics analysis in the presence or absence of PFA. (*D*) Whole cell lysates (Cell) or supernatants were digested with EndoH (E) or PNGaseF (P). Control (C) samples had no enzyme added. After overnight digestion, samples were separated on SDS-PAGE followed by western blot and stained for the V5 tag. (*E*) Cells were fixed, permeabilized, and stained for the indicated proteins before imaging by microscopy. (*F*) Whole cell lysates or supernatants were digested overnight with PNGaseF (P) or a mixture of PNGaseF and O-Glycosidase, along with Neuraminidase, Galactosidase S, and Acetylhexosaminidase (P+O). Control samples (C) had no enzymes added. Samples were then separated by SDS-PAGE and analysed by western blot for the V5 tag. (*G*) HCMV-infected cells, supernatants, or purified virions were analysed by SDS-PAGE followed by western blot and stained as indicated. All experiments are representative of at least three repeats.

An Adenoviral (Ad) vector library was generated, containing all 170 canonical HCMV genes, with each C-terminally tagged with a V5 epitope tag. When supernatants from infected cells were analyzed by western blot, the construct encoding UL4 was observed to release a substantial amount of epitope-tagged product into the supernatant. To ascertain whether the UL4 gene product is also secreted in the context of HCMV infection, a V5 tag was inserted in to the 3′ end of UL4 within the HCMV genome. Western blots revealed that UL4-V5 produced a protein with an apparent molecular mass of 50 kDa in both Ad-UL4- and HCMV-infected cells, while a slower migrating product (55–60 kDa) was detected in the supernatant (Fig. 1*A*). In a timecourse, UL4-V5 expression was detected in cells and supernatant from 72 h onward, with its abundance in the supernatant increasing with time (Fig. 1*B*). These expression kinetics are supported by proteomic data, which also showed UL4 to be a late gene product (temporal class 5; Tp5) whose expression is blocked by phosphonoformic acid (PFA), an inhibitor of virus DNA replication (Fig. 1*C*) (31).

UL4 is predicted to encode a 149 amino acid polypeptide with an eighteen amino acid signal sequence and a molecular mass of 17 kDa, yet also contains eight consensus N-linked glycosylation sites. Both the intracellular and extracellular forms of the expressed protein were reduced to more rapidly migrating species by treatment with Peptide:N-glycosidase F (PNGaseF); the UL4 gene product is thus a glycoprotein (gpUL4). The intracellular form of gpUL4-V5 was sensitive to endoglycosidase H (EndoH), whereas the slower migrating extracellular form had acquired resistance, consistent with the extracellular form being modified during transit through the Golgi apparatus prior to secretion (Fig. 1*D*). In accordance with the majority of the intracellular glycoform being immature, gpUL4-V5 colocalized with markers of the ER following expression both in isolation (RAd-UL4), and from HCMV (Fig. 1*E*).

PNGaseF digestion reduced the size of intracellular gpUL4-V5 to the mass predicted from its amino acid sequence (17 kDa). However, the PNGaseF-treated secreted form migrated with a mass of ~20 kDa (Fig. 1*D*). When treated with both O-Glycosidase and PNGaseF, the intracellular and secreted forms comigrated with a mass of 17 kDa, indicating the presence of O-linked glycosylation on the secreted form (Fig. 1*F*). The reduction in size of the secreted form from 50 to 60 kDa to 17 kDa following PNGaseF and O-glycosidase treatment is consistent with the presence of multiple N- and O-glycosylation sites, with carbohydrates amounting to >70% of the apparent mass of the secreted protein.

The UL4 protein was previously identified as a component of HCMV strain Towne virions (32), yet its presence was not reported in the proteome of strains AD169 or TB40 virions (33, 34). Since the genetic integrity of these laboratory strains have all been compromised during extensive in vitro passage, the issue of whether UL4 is part of the virion needed to be addressed using a virus containing a complete gene complement. To this end, HCMV strain Merlin virions were purified using glycerol tartrate gradient centrifugation. gpUL4-V5 could be detected in infected cells, and the supernatant from infected cells, but not in virions. In contrast, the matrix protein pp28 could be detected in infected cells, virions but not in the supernatant (Fig. 1*G*). These findings are consistent with gpUL4 being a secreted protein and not a virion component.

### UL4 Inhibits NK Cell Activation.

**Fig. 2.**
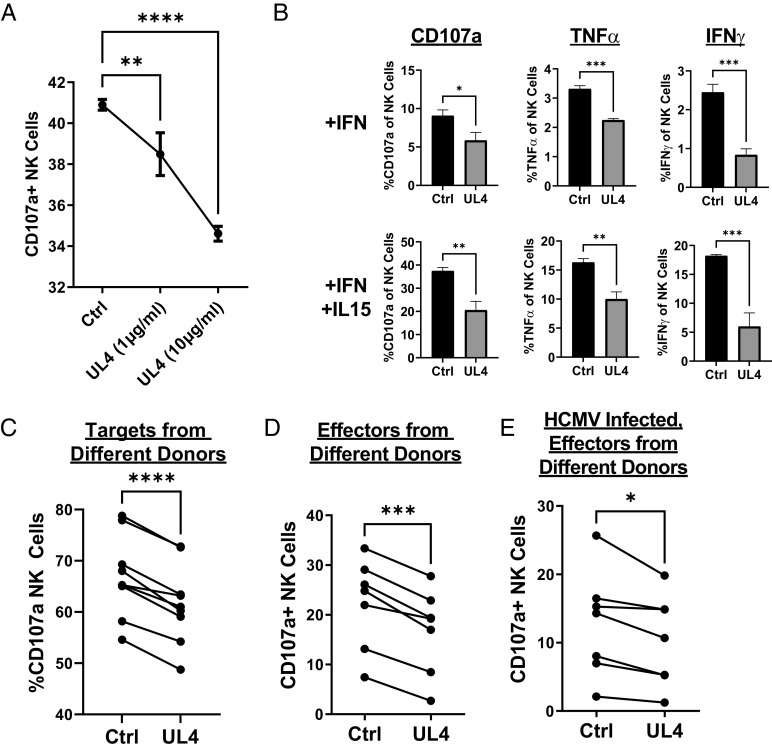
UL4 inhibits NK cell activation in a dose-dependent manner. Cells were either mock infected (*A*–*D*) or infected with HCMV strain Merlin (*E*) for 96 h. Targets were incubated with NK cells (*A* and *C*–*E*) or PBMCs (*B*) as effectors for 5 h, and the proportion of degranulating NK cells (CD3^−^CD56^+^) was assessed. Unless otherwise indicated, proteins were included in assays at 5 μg/mL. NK cells were prestimulated with IFNα (*A*–*E*), or IFNα and IL-15 (*B*). (*A*) Purified UL4-V5 protein was added into assays at the indicated doses. (*B*) In addition to degranulation, cells were permeabilized and stained for IFNγ and TNFα, and the proportion of cells expressing each cytokine was assessed. (*C*) Skin fibroblasts from 6 different donors, as well as HFFF from three different sources, were used as targets, along with NK cells from a single donor. (*D* and *E*) Mock (*D*) or HCMV infected (72 h postinfection) (*E*) HFFF-Terts were used as targets, along with PBMC from seven different donors. One-way ANOVA with Tukey correction (*A*), T test (*B*), and paired *t* test (*C*–*E*): *P < 0.05, **P < 0.01, ***P < 0.001, and ***P < 0.0001. All experiments are representative of at least three repeats.

NK cells are critical to the control of HCMV, and HCMV dedicates a substantial portion of its coding capacity to evading NK cell recognition. In addressing the function of UL4, we therefore investigated its impact on NK cell activation. gpUL4-V5 was purified from the supernatant of cells infected with the Ad-UL4 recombinant. Interferon-stimulated NK cells were incubated with an uninfected target cell population, and NK cell degranulation was assessed in a CD107a mobilization assay. gpUL4-V5 suppressed NK cell activation in a dose-dependent manner (Fig. 2*A*). Using a similar experimental setup, gpUL4-V5 also inhibited IFNγ and TNFα induction by NK cells, even when the effectors were super-stimulated with both IFNα and IL15 (Fig. 2*B*). UL4-mediated inhibition of NK cell activation was evident using dermal fibroblast targets from multiple donors (Fig. 2*C*) and also effector cells (PBMCs) from multiple donors (Fig. 2*D*). gpUL4-V5 was therefore able to suppress NK cell activation in a donor-independent manner. In addition, gpUL4-V5 was capable of inhibiting NK activation when HCMV-infected cells were used as targets, although the magnitude of this effect was much smaller than with uninfected cells (Fig. 2*E*), consistent with viral expression of multiple NK cell evasion functions.

**Fig. 3.**
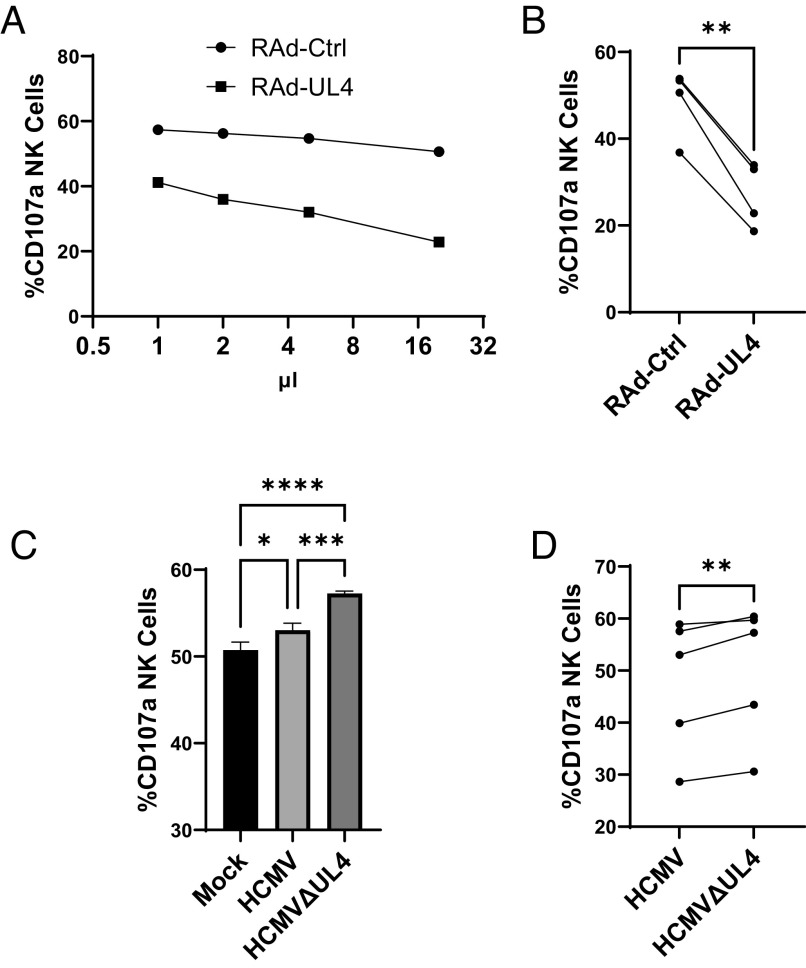
Untagged UL4 inhibits NK cell degranulation in isolation and in the context of infection. Supernatant was harvested from cells infected with RAd expressing untagged UL4, or empty control vector (*A* and *B*) or with Mock infected, HCMV, or HCMV deleted for the UL4 ORF (HCMVΔUL4; *C* and *D*) and concentrated 20-fold. NK cells were then prestimulated with IFNα and IL-15 before being incubated with HFFF for 5 h in the presence of the indicated supernatants (*A*), or with 5 µL of supernatant (*B*–*D*), then assessed for degranulation (CD107a). PBMCs from a single donor (*A* and *C*) or multiple donors (*B* and *D*) were used. One-way ANOVA with Tukey correction (*C*) and paired *t* test (*B* and *D*): *P < 0.05, **P < 0.01, ***P < 0.001, and ***P < 0.0001.

To determine whether the V5 tag affected the function of gpUL4, untagged wild-type gpUL4 was harvested from the supernatant of cells infected with an Ad vector or HCMV strain Merlin. When tested in CD107a assays, the supernatant from RAd-UL4 was capable of inhibiting NK-cell activation in a dose-dependent manner (Fig. 3*A*) from multiple donors (Fig. 3*B*). Similarly, HCMV-infected cell supernatants stimulated NK degranulation more strongly when harvested from a strain lacking the UL4 ORF, as opposed to one expressing it (Fig. 3 *C* and *D*). The V5-tag does not appear to impact the function of gpUL4.

### gpUL4 Binds TRAIL with High Affinity.

**Fig. 4.**
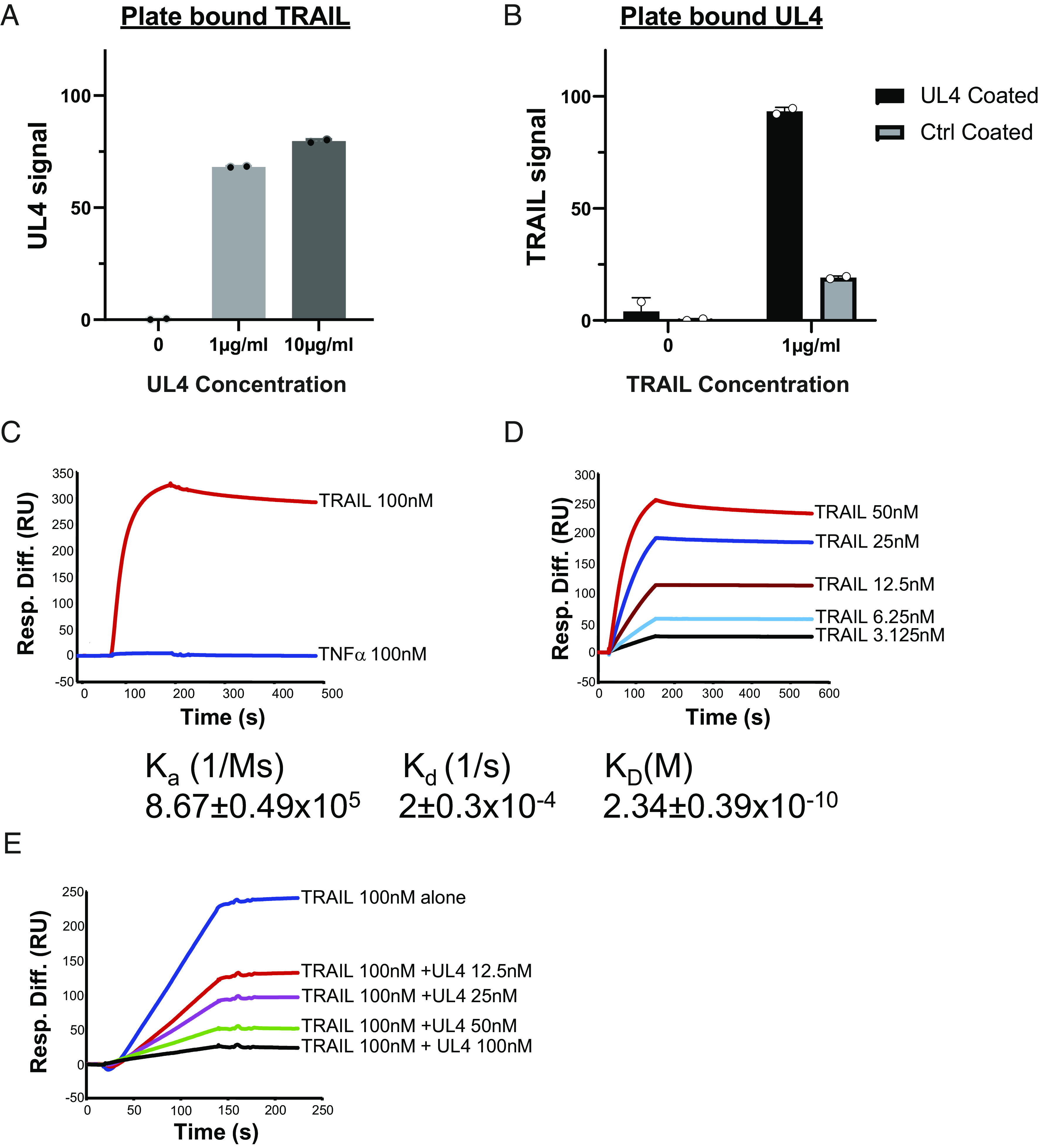
UL4 binds to TRAIL with high affinity. (*A*) Plates were coated with TRAIL, and the amount of gpUL4-V5 binding was assessed by ELISA. (*B*) Plates were coated with either gpUL4-V5 or control protein, and the amount of TRAIL binding was assessed by ELISA. (*C*) TRAIL (100 nM) or TNFα (100 nM) binding to gpUL4-V5 was analyzed by surface plasma resonance (SPR) of binding curves subtracted for binding to a control surface (soluble UL141). (*D*) TRAIL binding kinetics and affinity measurements were examined using increasing TRAIL concentrations. The mean on-rate (K_a_), off-rate (K_d_), and affinity (K_D_) plus or minus the SEM of the results from three independent experiments are indicated. (*E*) The effect of UL4 on the binding of TRAIL to TRAIL-R1 (test surface) subtracted background from the control surface (soluble UL141) was examined. Increasing concentrations of gpUL4-V5 were preincubated with 100 nM TRAIL before being flowed over the chip. Experiments are representative of at least three repeats.

We recently deployed mass spectrometry to identify the intracellular interacting partners for the products of all 170 canonical HCMV genes in a productive infection (35). Interactions were assessed by multiple criteria; however, TRAIL (TNFSF10) consistently scored as the highest probability interactor for gpUL4-V5 (Table 1). To test the validity of this interaction, an ELISA assay was performed. gpUL4-V5 was observed to bind to TRAIL-coated plates in dose-dependent fashion (Fig. 4*A*). In the inverse experiment, TRAIL also bound to plates coated with gpUL4-V5 (Fig. 4*B*). Surface Plasmon Resonance (SPR) was performed to confirm and quantify the strength of the interaction (Fig. 4 *C* and *D*). TRAIL, but not TNFα, bound to gpUL4-V5 coated onto the chip (Fig. 4*C*), with a kinetics analysis indicating a fast on-rate of 8.67 × 10^5^ 1/Ms, a slow off-rate of 2 × 10^−4^ 1/s and an affinity of 234 pM (Fig. 4*D*). gpUL4-V5 thus binds TRAIL with high affinity. Importantly, the binding of gpUL4-V5 to TRAIL inhibited TRAIL from binding its cognate receptor TRAILR1 (TRAIL receptor 1/TNFRSF10A) in a dose-dependent manner (Fig. 4*E*). gpUL4 thus acts as a decoy receptor for soluble TRAIL.

**Table 1.** Interactome data for UL4, taken from ref. 35

Prey species	Prey gene name	Prey uniprot	Average PSMs	Entropy	z-score	NWD score
HUMAN	TNFSF10	P50591	1.5	0.95	12.33	53.54
HUMAN	STC2	O76061	2	0.88	11.02	22.98
HCMVM	UL14	Q6SWB7	1.5	0.95	0.46	6.73
HCMVM	UL116	Q6SW34	2.5	0.98	1.15	4.70
HCMVM	UL130	F5HCP3	2	1.00	0.66	4.57
HUMAN	FAT1	Q14517	2.5	0.81	1.08	3.37
HCMVM	UL4	Q6SWC6	3	0.94	8.75	2.62
HUMAN	FBXO2	Q9UK22	3.5	0.90	5.61	2.30
HUMAN	CNNM3	Q8NE01	2.5	0.98	1.37	2.27
HUMAN	SLC25A22	Q9H936	2.5	0.98	3.80	1.41
HCMVM	UL75	Q6SW67	13.5	1.00	1.41	1.08
HUMAN	MOCS3	O95396	1.5	0.95	2.88	1.03
HCMVM	UL47	Q6SW85	8.5	1.00	0.13	1.01

Data reported here include a) the number of peptide spectral matches (PSMs), averaged between technical replicates; b) an entropy score, which compares the number of PSMs between replicates to eliminate proteins that are not detected consistently; c) a z-score, calculated in comparison to the average and SD of PSMs observed across all Ips; and d) a normalized WD (NWD) score. The NWD score addresses whether (i) the protein is detected across all Ips and (ii) whether it is detected reproducibly among replicates. It was calculated using the fraction of runs in which a protein was observed, the observed number of PSMs, the average and SD of PSMs observed for that protein across all Ips, and the number of replicates (1 or 2) containing the protein of interest. NWD scores were normalized so that the top 2% earned scores of ≥1.0.

### UL4 Inhibits TRAIL-Mediated Killing.

**Fig. 5.**
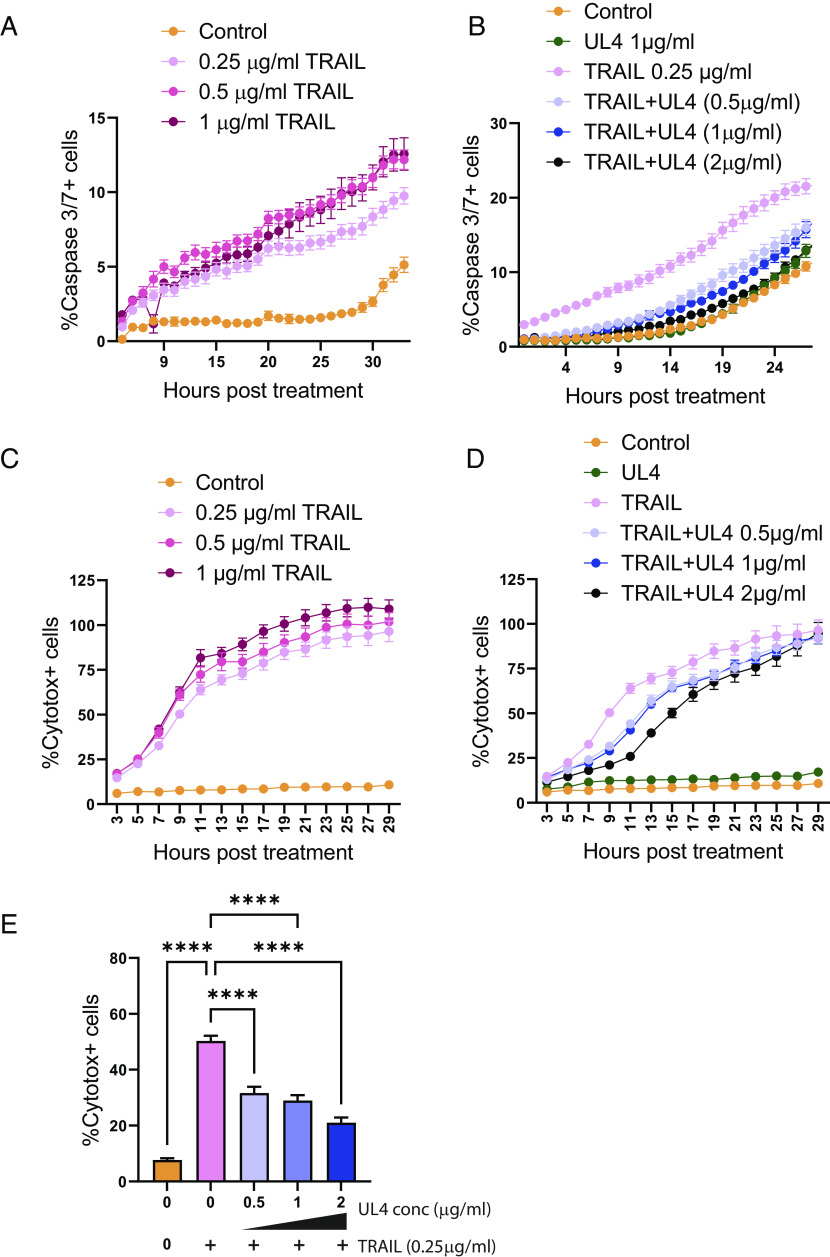
UL4 inhibits TRAIL-mediated death of target cells. (*A* and *B*) HFFF-TERT cells were incubated with the indicated proteins and analysed over time for the level of Caspase 3/7 activation by Incucyte. (*C* and *D*) HFFF-Tert were incubated with the indicated proteins, and the level of cytotoxicity was assessed over time by Incucyte. (*E*) Summary of the 9-h timepoint from (*D*). One-way ANOVA with Tukey correction: *P < 0.05, **P < 0.01, ***P < 0.001, and ***P < 0.0001. Experiments are representative of at least three repeats.

TRAIL is a member of the TNF superfamily that exists in soluble and membrane-bound forms. Both forms can bind the death receptors TRAILR1 (TNFRSF10A) and TRAILR2 (TNFRSF10B) to stimulate the extrinsic apoptotic pathway (36). In the SPR assay, gpUL4-V5 impeded the TRAIL:TRAIL-R1 interaction. We therefore sought to investigate whether gpUL4 could inhibit TRAIL-induced apoptosis in a functional assay. Human fibroblasts express both TRAILR1 and TRAILR2 (37); HFFFs were therefore treated with cycloheximide to generate a proapoptotic state before being incubated with soluble TRAIL. The induction of apoptosis was assessed in real time using a fluorogenic caspase 3/7 assay as well as Cytotox Red uptake; dead cells take up the dye when the integrity of the plasma membrane is compromised. TRAIL induced substantial levels of caspase 3/7 activation (Fig. 5*A*) and loss of membrane integrity (Fig. 5*B*) in a dose-dependent manner with both effects efficiently inhibited in the presence of gpUL4-V5 (Fig. 5 *C*–*E*). gpUL4 was therefore able to suppress the capacity of soluble TRAIL to induce apoptotic cell death.

### UL4 Inhibits NK Cell Activation through Binding to Membrane-Bound TRAIL.

**Fig. 6.**
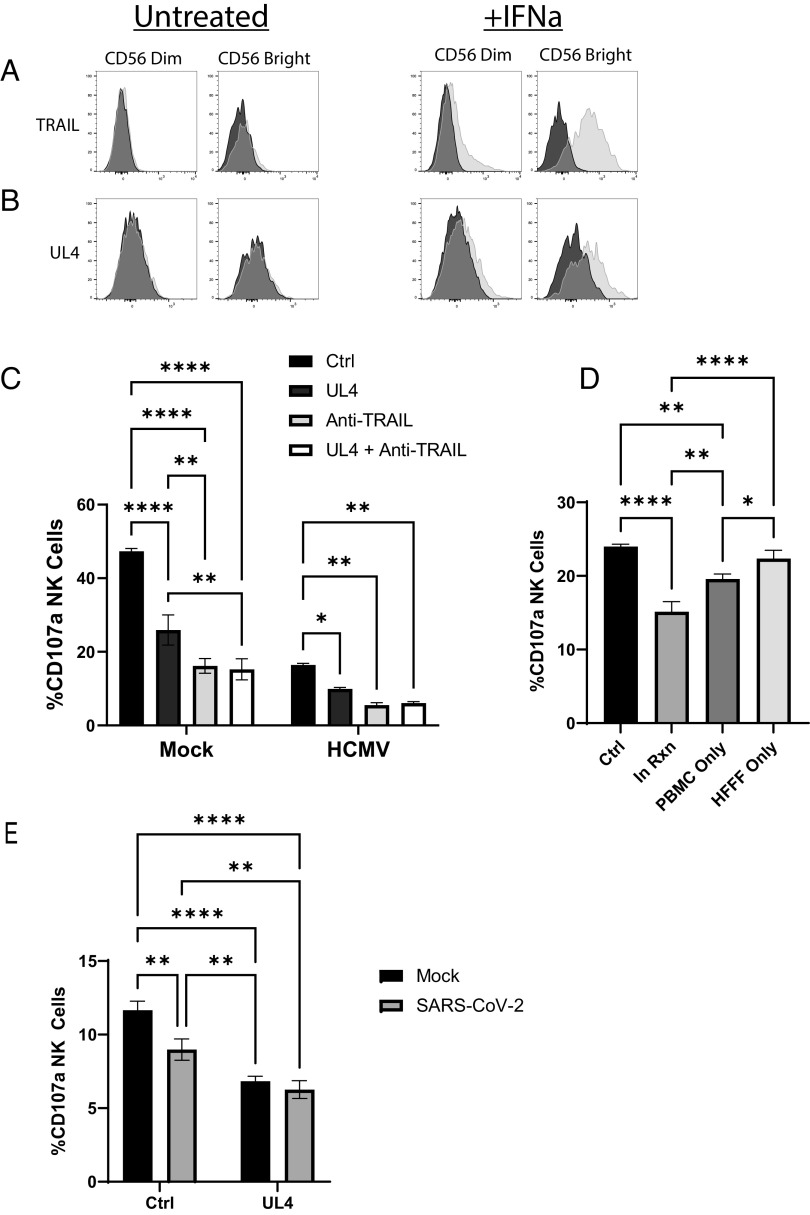
UL4 binds and inhibits NK cells in a TRAIL-dependent manner. (*A*) PBMCs were stimulated overnight with IFNα, or unstimulated, then stained for CD3/CD56 along with either TRAIL (*A*) or gpUL4-V5 (*B*), before gating on the CD3^−^CD56^+^ population, and plotting the level of TRAIL or UL4 staining. (*C*) Mock or HCMV-infected HFFF-hTert were incubated with PBMC for 5 h in the presence of the indicated proteins (5 μg/mL); then, the level of degranulating NK cells (CD3^−^CD56^+^) was assessed. (*D*) NK cells and HFFF-hTert were mixed in the presence of gpUL4-V5 (5 μg/mL) protein. gpUL4-V5 was kept throughout the entire reaction (in Rxn), or PBMCs were pretreated with gpUL4-V5 and excess washed off (PBMC only), or HFFF-hTert were pretreated with protein and excess washed off (HFFF only), then the level of degranulating NK cells (CD3^−^CD56^+^) assessed 5 h later. (*E*) A549-ACE2 targets infected with SARS-CoV-2 for 24 h were coincubated with NK cells for 5 h; then, the level of degranulating NK cells (CD3^−^CD56^+^) was assessed. Two-way ANOVA with Tukey correction (*C* and *E*) and one-way ANOVA with Tukey correction (*D*): *P < 0.05, **P < 0.01, ***P < 0.001, and ***P < 0.0001.

We next assessed whether the NK cell suppressive function associated with gpUL4 was mediated by its interaction with TRAIL. TRAIL has been reported as absent from NK cells that are directly isolated from blood yet inducible by treatment with IL-2, IL-15, or IL-12 (38–40). TRAIL was indeed not detectable on NK cells in PBMCs analyzed directly from our donors but was induced across both CD56^bright^ and CD56^dim^ populations following stimulation with IFNα (Fig. 6*A*). When soluble gpUL4-V5 was added to these cell populations and stained, gpUL4-V5 binding was limited to the IFNα-activated populations, with both TRAIL expression and gpUL4-V5 binding being higher on CD56^bright^ compared to CD56^dim^ populations (Fig. 6*B*).

Functional assays were therefore performed using IFNα-stimulated NK cells. NK cells were activated more by mock-infected than HCMV-infected cells, an effect attributable to HCMV’s immune evasion functions. gpUL4 secreted by HCMV-infected cells was washed away with media changes during experimental setup. Exogenous gpUL4-V5 was therefore added and proved capable of suppressing NK cell activation induced by both uninfected and HCMV-infected cells (Fig. 6*C*). Treatment with either a TRAIL-blocking antibody or gpUL4-V5 led to an inhibition of NK degranulation; however, the effect of combining the TRAIL-blocking antibody and gpUL4-V5 together was not additive. These findings are consistent with both gpUL4 and the TRAIL blocking antibody inhibiting NK activation through binding the same target.

TRAIL exists in both membrane-bound and soluble forms. gpUL4 can act as a decoy receptor for soluble TRAIL (Fig. 6). To investigate whether gpUL4 was capable of also targeting membrane-bound TRAIL, an NK degranulation assay was carried out in which only NK cells were pretreated with gpUL4-V5 and excess removed by washing cells (Fig. 6*D*). gpUL4-V5 was capable of inhibiting NK activation when NK cells were precoated but not when the target cells were pretreated. Thus, gpUL4 acts directly on NK cells, with at least some of this activity due to targeting of membrane-associated TRAIL.

### UL4 Suppresses the NK Cell Response to a Heterologous Virus.

UL4 has the capacity to act as an immune evasin in the context of HCMV infection. However, as a secreted protein, gpUL4 has further potential to be immunosuppressive in the context of a secondary infection. To explore this possibility, gpUL4-V5 was added directly to an NK assay with SARS-CoV-2 infected target cells. We have previously shown that SARS-CoV-2 suppresses the expression of NK cell activating ligands as part of a generalised inhibition of host transcription and translation (41). Thus, SARS-CoV-2-infected cells are less capable of stimulating NK cell activation than uninfected cells, although this evasion strategy was weaker than that seen with HCMV-infected cells (Fig. 6*E*). Importantly, however, gpUL4-V5 was able to further suppress the NK cell response to SARS-CoV-2-infected cells. This illustrates the potential for gpUL4 secreted from HCMV-infected cells to have additional immune consequences through systemic modulation of host immunity.

## Discussion

Previously characterised HCMV functions that suppress NK and T cell immunity act on proteins expressed on the infected cell surface. In contrast, UL4 was found to encode a protein that is secreted from HCMV-infected cells and not a structural component of HCMV virions. As a secreted function, gpUL4 has the potential to influence both bystander cell apoptosis and NK activation much more broadly, as evidenced by its effects on NK responses to SARS-CoV-2.

TRAIL induces apoptosis via the extrinsic pathway following binding to TRAILR1 and TRAILR2 as a homotrimer (42, 43), which in turn recruits Fas-associated death domain (FADD), followed by pro-caspase-8 and pro-caspase-10, which are then cleaved and activated (44, 45), ultimately leading to apoptosis. TRAIL is thought to be an important component of antiviral immunity (46)–indeed HCMV impedes TRAIL-mediated apoptosis at other stages of the pathway: UL141 down-regulates TRAILR1 and TRAILR2 from the infected cell surface (37) while UL36 inhibits cleavage and activation of pro-caspase 8 within the infected cell (47). Although virus-encoded soluble TRAIL inhibitors have not been previously described, antagonism of related TNF-superfamily pathways is common. HCMV also targets Fas/CD95 through an unknown mechanism (48), mouse CMV targets TRAIL receptors via m166 (49), and multiple poxviruses encode soluble TNF-alpha receptors (50–55). These observations demonstrate the importance of TNF superfamily pathways to viral control. HCMV is not the only virus that produces a secreted protein capable of modulating NK function, the orthopoxvirus MHC class I–like protein (OMCP) is a soluble NKG2D agonist (56) and the owl monkey CMV CD48 homologue suppresses 2B4-mediated NK-cell activation (57). We now show that gpUL4 also acts as a soluble decoy receptor for TRAIL. The strength of the binding affinity of gpUL4 to TRAIL (234 pM) was comparable to that of a high-affinity antibody and ~8- to ~300-fold higher than the affinity of TRAIL for its receptors TRAIL-R1 (70 nM) and TRAIL-R2 (2 nM) (58). UL4 has the capacity to protect HCMV-infected cells not only from apoptosis-from-without mediated by soluble TRAIL but also from membrane-bound TRAIL expressed on cytotoxic effector cells.

TRAIL is absent from resting NK cells but is up-regulated in response to interferon, IL-12 or IL-15. While TRAIL activity on NK cells has previously been associated with its proapoptotic role, in which membrane-bound TRAIL engages TRAILR on the target cell to induce apoptosis (39, 40, 59–61), here UL4’s function was mapped through its capacity to suppress TRAIL-dependent NK cell degranulation and cytokine secretion. Moreover, NK degranulation was impaired by pretreatment of membrane-bound TRAIL on NK cells prior to the assay. Our results with gpUL4 thus imply that membrane-bound TRAIL plays a regulatory role in NK cell activation that is distinct from its proapoptotic function. This is unexpected as TRAIL has neither intracellular immunoreceptor tyrosine-based activation motifs nor positively charged amino acids in its transmembrane domain to aid recruitment of adapter molecules and facilitate downstream signalling. However, it is consistent with a recent report demonstrating that TRAIL cross-linking induces NK degranulation (62).

The HCMV genome contains 15 gene families that range in size from 2 to 14 members. The RL11 family is the largest and consists primarily of a long tandem gene array clustered at one end of the U_L_ gene region that likely arose via an ancient accordion gene expansion (*SI Appendix*, Fig. S1). RL11 gene families are found in the cytomegaloviruses of the Old World, but not the New World monkeys, and thus may have been acquired 25 to 35 Mya (30, 63). The RL11 family members have diverged in their various old-world monkey hosts (30, 63, 64), with the closest equivalents to the HCMV genes most clearly observed in chimpanzee CMV (*SI Appendix*, Fig. S1) (65). In HCMV, the RL11 gene family is associated with a series of immunological functions: RL11 and RL13 each encode IgG-binding proteins (66, 67), UL7 encodes an FLT3 ligand (68), UL8 an inhibitor of cytokine secretion (69, 70), UL10 an inhibitor of T cell activation, and UL11 targets CD45 (71–73). Following expression as surface glycoproteins, the ectodomains of UL7, UL8, and UL10 can be released into the supernatant following proteolytic cleavage, although in the case of UL7 and UL8 this is prevented during infection because HCMV inhibits the activity of the metalloprotease responsible (ADAM17) (74). Interestingly, Adenovirus type 19a also contains a single RL11 homologue which is capable of suppressing NK and T cell function via a direct interaction with CD45 and can also be released from cells by proteolytic cleavage (75). While a UL4 gene has been designated within chimpanzee CMV (*SI Appendix*, Fig. S1), it is a positional rather than a sequence homologue that appears to have originated from a duplication of a UL5 homologue (*SI Appendix*, Fig. S2). Although it is a member of the RL11 gene family, HCMV UL4 may therefore have been functionalised after chimpanzee–human divergence 4 to 11 Mya and provides an example of a virus-encoded soluble TRAIL antagonist.

gpUL4 is highly glycosylated (both N- and O-linked), which may act both to enhance its stability and protect it from antibody recognition. A subset of HCMV RL11 family members exhibit such an extraordinary level of interstrain sequence variation that they are considered “hypervariable” (RL6, RL11, RL13, UL1, UL9, and UL11). Although relatively conserved, gpUL4 nevertheless exhibits a significantly higher sequence variation across clinical isolates than most other HCMV proteins (76, 77), with isolates exhibiting as little as 76% sequence identity, and sequences clustering into seven distinct genotypes (*SI Appendix*, Fig. S3). Thus, if antibody is induced to gpUL4 during an infection, it may not necessarily neutralise gpUL4 from a super-infecting variant.

UL4 is transcribed with early phase kinetics (32), yet its protein does not appear until the late phase. UL4 is known to be subject to posttranscriptional control that delays its expression: the antiviral restriction factor ZAP-1 binds UL4 mRNA to promote its degradation (78) while an upstream ORF (µORF2) within the 5′ leader of the mRNA impedes its translation (79). Furthermore, the UL4 ORF is contained in three separate transcripts that are expressed from different promoters at different stages of the infectious cycle, although it remains unclear whether all three transcripts can produce the UL4 protein (80). How µORF2 regulates expression in response to different cell types and environments is unknown, nor is it yet clear how deploying such complex mechanisms to regulate UL4 expression benefits the virus.

gpUL4 is a viral-encoded soluble inhibitor of TRAIL, inhibits both TRAIL-induced apoptosis and TRAIL-induced NK activation, and has broader immunosuppressive effects. HCMV establishes a lifelong chronic infection characterised by repeated periodic reactivation, during which gpUL4 may be released at levels dependent on both the latent virus load and the efficacy of reactivation. Tracking levels of the secreted UL4 protein in vivo could provide a useful biomarker of HCMV reactivation during asymptomatic carriage and/or overt disease. In vivo measurements could also inform on if, and when, gpUL4 is produced in levels sufficient to promote an immunosuppressive environment.

## Methods

### Cells and Viruses.

Telomerase immortalized human fetal foreskin fibroblasts (HFFF-hTert) and versions expressing the coxsackie-adenovirus receptor (HFFF-CAR) have been described previously (81, 82). hTert immortalized skin fibroblasts (Sfi) were isolated from healthy donors following punch biopsy. A549 cells expressing the ACE2 receptor for SARS-CoV-2 have been described previously (41). All cells were maintained in modified eagle’s medium (DMEM) containing 10% fetal calf serum (FCS) at 37 °C and 5% CO_2_. SARS-CoV-2 strain England2 was a gift from Public Health England and has a genome identical to the isolate initially recovered from Wuhan. HCMV strain Merlin was recovered from a bacterial artificial chromosome (BAC) clone known as pAL1111 as previously described (83) and contains a complete wild-type genome with the exception of disrupting mutations in RL13 and UL128, which are required to ensure stability during in vitro propagation in fibroblasts (84, 85). Recombineering was used to modify virus genomes as previously described (83); a C-terminal V5 tag was engineered into the Merlin genome using primers (​AGA​TGT​GAA​TTC​CAC​CTT​TTA​TTA​CTC​TTG​TTA​CAA​CCT​GAC​CGT​GAC​CAG​CGC​TGG​TAA​GCC​AAT​CCC​TAA​CCC​GCTCCTAGGTCTTGATTCTACGTAA, G​TAA​ACA​CAA​TAG​CTA​CAG​CCA​CGC​GGC​TCT​GTG​GAA​CTT​TAC​ATG​CGT​TTA​CGT​AGA​ATC​AAG​ACC​TAG​GAG​CGG​GTTAGGGATTGGCTTACCAGCGCT), the UL4 ORF was deleted from the viral genome using primers (​GTT​CCT​TTT​TTT​TTG​TTT​TTG​CAT​CAC​TTA​TCG​CCA​CTA​TCA​GTG​CAA​TAT​TTT​GAT​TGT​GAG​ACT​GAA​AGA​GTA​TCGTTCCTGTGACGGAAGATCACTT, C​GTA​GGG​ACG​CTT​AAC​ATT​TCA​AAA​GCA​ACG​TAA​ACA​CAA​TAG​CTA​CAG​CCA​CGC​GGC​TCT​GTG​GAA​CTT​TAC​ATG​CGTCTGAGGTTCTTATGGCTCTTG), and UL4 lacking a epitope tag was cloned into the pAdZ-5 vector (29) using primers G​TCA​GAT​CGC​CTG​GAG​ACG​CCA​TCC​ACG​CTG​TTT​TGA​CCT​CCA​TAG​AAG​ACA​CCG​GGA​CCG​ATC​CAG​CCT​GGA​TCCATGATGCTTAGAACGTGGATATCA and G​GCG​TGA​CAC​GTT​TAT​TGA​GTA​GGA​TTA​CAG​AGT​ATA​ACA​TAG​AGT​ATA​ATA​TAG​AGT​ATA​CAA​TAG​TGA​CGT​GGG​ATCCTTAGGTCACGGTCAGGTTGT. For all experiments, cells were infected at MOI = 5 for 2 h in a small volume with rocking.

### Protein Production.

The coding sequence of UL4 from HCMV strain Merlin was cloned into a replication-deficient Adenovirus vector (RAd) by recombineering as previously described (29). The UL4 gene was synthesized to contain a C-terminal V5/avitag/6his epitope tag for purification and detection. A vector expressing the epitope tag alone was generated in a similar manner and used as a negative control. To produce protein, HFFF-CAR were infected at MOI = 200, in the absence of FCS but with 10 μM forskolin, and media were collected 3 and 6 d later. Proteins were purified on an AKTA using a His-Trap column and in some cases purified further by size-exclusion chromatography. We also generated supernatants from Ad vectors expressing untagged UL4 in which HF-CAR cells were infected at MOI = 200 and from HFFF infected with HCMV or HCMV-ΔUL4 at MOI = 5. In both cases, media containing FCS were exchanged for media lacking FCS at 24 h postinfection. Media were collected at 8 d postinfection, cell debris was removed by centrifugation, and samples were concentrated 20-fold using a Vivaspin (Sartorius) with 10-kDa MWCO. Samples were diluted into the supernatant of the experiment to a final 1× concentration (i.e., 5 µL in 100).

### Virion Purification.

HCMV virions were harvested from the supernatant of infected HFFF-hTert when 100% CPE was reached. Cells were pelleted by a low-speed spin (1,500 rpm, 5 min); then, virus was pelleted from the supernatant by centrifuging at 14,000 rpm for 2 h in a JLA-16.25 rotor. Virus was resuspended and purified away from cellular debris, dense bodies, and noninfectious enveloped particles using a positive density/negative viscosity glycerol tartrate gradient as previously described (86).

### Western Blot and Deglycosylation.

Samples were lysed in 1 × LDS sample buffer (ThermoFisher) containing 10% 1M dithiothreitol (DTT) and boiled at 100 °C for 10 min before being loaded onto a 10% Bis-Tris gel (Bio-Rad). After separation for 40 min at 200 V, proteins were transferred to PVDF in a semidry blotter at 20 V for 1 h. Membranes were blocked in blocking buffer [5% nonfat milk in PBS containing 0.1% Tween (PBST)] for 1 h. Primary antibody was added in blocking buffer for 1 h, washed, and secondary antibody [anti-mouse or anti-rabbit IgG conjugated to horseradish peroxidase (Bio-Rad)] added in blocking buffer for 1 h. After washing, membranes were reacted with SuperSignal West Pico (ThermoFisher) and imaged on a Syngene XX6. Primary antibodies were mouse anti-V5 (AbD Serotec), or mouse anti-GAPDH (Abcam), rabbit anti-actin (Sigma-Aldrich) or HCMV pp28 (clone 5C3, Santa Cruz). For deglycosylation, samples were treated with either EndoH or PNGaseF alone or with Protein Deglycosylation mix II, which additionally contains O-glycosidase enzymes, for 1 h overnight according to the manufacturer’s instructions (New England Biolabs), before loading onto protein gels as above.

### Immunofluorescence.

Cells were plated on coverslips before being infected with either HCMV or RAd vectors. At appropriate times, cells were fixed in 4% paraformaldehyde and permeabilized in 0.5% NP-40. Coverslips were incubated with primary antibody at 37 °C for 30 min, washed, and then incubated with secondary antibody along with DAPI in the same manner before being mounted in DABCO mounting medium. Slides were imaged on a Zeiss Axio Observer Z1 with Apotome 2. Primary antibody was mouse anti-V5 and rabbit anti-calnexin (Abcam); secondary antibodies were anti-mouse Alexa Fluor 488 and anti-rabbit Alexa Fluor 594 (ThermoFisher).

### NK Activation Assays.

Peripheral blood mononuclear cells (PBMCs) were collected from healthy donors and separated on Histopaque. PBMCs were either rested or incubated with interferon α (1,000 IU/mL), overnight. On the day of the assay, 50,000 targets were incubated with 500,000 PBMCs in the presence of GolgiStop (BD) and CD107a-FITC antibody (BioLegend) in a total volume of 100 µL. After 5 h, cells were stained with live/dead aqua (ThermoFisher), CD56-BV605 (BioLegend), and CD3-PE-Cy7 (BioLegend). After being washed, cells were fixed in 4% PFA and analysed on an Attune Cytometer (ThermoFisher). Cells were gated on live NK cells (CD3−/CD56+), and the percentage of CD107a was assessed. In some assays, intracellular cytokine staining was performed. In this case, Brefeldin A was included in the assay, and following cell surface staining, cells were fixed and permeabilized (BD Fix/Perm kit) and stained with TNFα-BV421 and IFNγ-APC.

### Incucyte Analysis.

A total of 10,000 HFFF-hTert cells per well were seeded in a microscopy grade, 96-well-plate (Ibidi), with DMEM F12 medium containing 10% FBS. Cells were treated with 5 μg/mL cycloheximiDe, TRAIL (Recombinant Human TRAIL, BioLegend), and/or UL4 (produced as described above) at various concentrations. Apoptosis was assessed by caspase 3/7 activation with CellEvent™ Caspase-3/7 Green Detection Reagent (Thermo Scientific). Cell death was assessed using Incucyte® Cytotox Red Dye (Sartorius) according to the manufacturer’s specifications. On both occasions, the signal was collected every 2 h for 48 h with an IncuCyte® ZOOM System (Sartorius).

### ELISA.

Qualitative ELISA was performed by coating the plate with either UL4 or control protein and measuring signal from the binding of TRAIL. Alternatively, the plate was coated with TRAIL (cat.no 752902, BioLegend), and signal was measured from the binding of gpUL4-V5. For coating, 1 μg/mL was used for each protein. The proteins were diluted in coating buffer pH 9.6 (Thermo), added on ELISA plates (MaxiSorp, Immulon 4HBX, Thermo), and incubated at 4 °C overnight. Next, the coating solution was removed, and the plate was washed with PBS followed by 2 h incubation at room temperature with blocking buffer (PBS, 0.05% Tween 20, 1% BSA). After blocking, the plate was washed with PBS-T (PBS, 0.05% Tween 20) and incubated with 1 or 10 μg/mL of UL4 or TRAIL for 90 min at 37 °C followed by washes with PBS-T. Primary antibody in blocking buffer was added for 1 h at room temperature, washed with PBS-T and secondary antibody in blocking buffer was added for another 1 h at room temperature followed by washes with PBS-T. Primary antibodies used were mouse anti-V5 antibody (Bio-Rad) or mouse anti-TRAIL (cat.no ab10516, Abcam), and the secondary antibody was goat anti-mouse HRP (Santa Cruz). Last, TMB Substrate Solution (Thermo) was added for 15 min at room temperature, and stop solution (Thermo) was added to stop the reaction. The absorbance was measured at 450 nm on an iMark absorbance microplate reader (Bio-Rad).

### Surface Plasmon Resonance (SPR).

SPR analysis was carried out on a Biacore T100 instrument (Cytiva). Control protein (soluble UL141, control flow cell, Fc1) or UL4 (test flow cell, Fc2) in acetate buffer pH 4.0 or pH 4.5, respectively (both Cytiva), was immobilized to a CM5 Series S chip (Cytiva) using the Biacore amine coupling kit (Cytiva) to an immobilized level of 5000RU. TNFα (PeproTech or BioLegend) or TRAIL (TNFRSF10A, BioLegend) were flowed over the chip in Biacore running buffer (10 mM HEPES, 150 mM NaCl, and 0.005% TWEEN-20, pH 7.4) at various concentrations and binding examined by using curves subtracting the control surface binding from the test surface binding (Fc2-Fc1). The chip surface was regenerated using diethylamine pH 11.9 (1:200 in dH_2_O). TRAIL binding kinetics and affinity was determined using a kinetics wizard and fitting the data from 3.125, 6.25, 12.5, 25, and 50 nM binding curves with a 1:1 binding fit.

In other experiments, control protein (soluble UL141; control flow cell, Fc3) and TRAIL-R (TNFRSF10A, Biovision, test flow cell, Fc4) in acetate buffer pH4.0 and pH5.0, respectively (Cytiva), were immobilized to a CM5 Series S chip using the Biacore amine coupling kit to an immobilized level of 5000RU. TRAIL was then flowed over the chip at 100 nM either alone or preincubated with increasing concentrations of UL4 (12.5 nM to 100 nM). Curves were used subtracting the control surface binding from the test surface binding (Fc4-Fc3).

### Alignment and Assignment of Genotypes.

All UL4 protein sequences were downloaded from GenBank, and a set of unique UL4 proteins were aligned using CLC Main. IQ-TREE2 was used to reconstruct a maximum likelihood tree (87) based on the best substitution model determined by ModelFinder (88). Branch support was determined using the ultrafast bootstrap with 1,000 replicates (89). Genotypes were defined using ClusterPicker (90) using a support threshold for cluster support of 0.9 and a genetic distance threshold of 0.1.

### Ethical Approval.

Healthy adult volunteers provided blood and dermal fibroblasts for this study after giving written informed consent in accordance with the principles of the Declaration of Helsinki. The study was approved by the Cardiff University School of Medicine Research Ethics Committee (reference numbers 10/20 and 16/52).

## Supplementary Material

Appendix 01 (PDF)

## Data Availability

All study data are included in the article and/or *SI Appendix*.
