# Supplementary material for: HCMV-secreted glycoprotein gpUL4 inhibits TRAIL-mediated apoptosis and NK cell activation
Source: Proc Natl Acad Sci U S A. 2023 Nov 27;120(49):e2309077120. doi: 10.1073/pnas.2309077120 (PMC10710050; doi:10.1073/pnas.2309077120)
Supplement: Supplementary file 1 — Appendix 01 (PDF) [file pnas.2309077120.sapp.pdf]

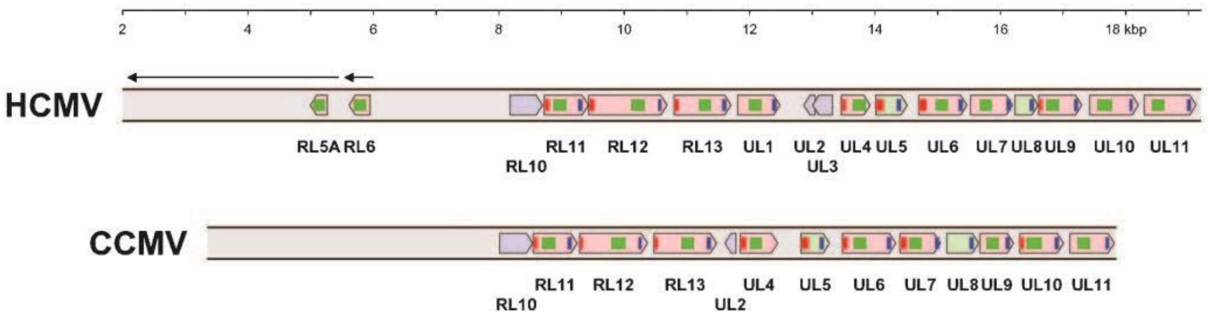

Figure S1. Comparison of the UL1 gene family in human and chimpanzee CMV. Taken from[30].

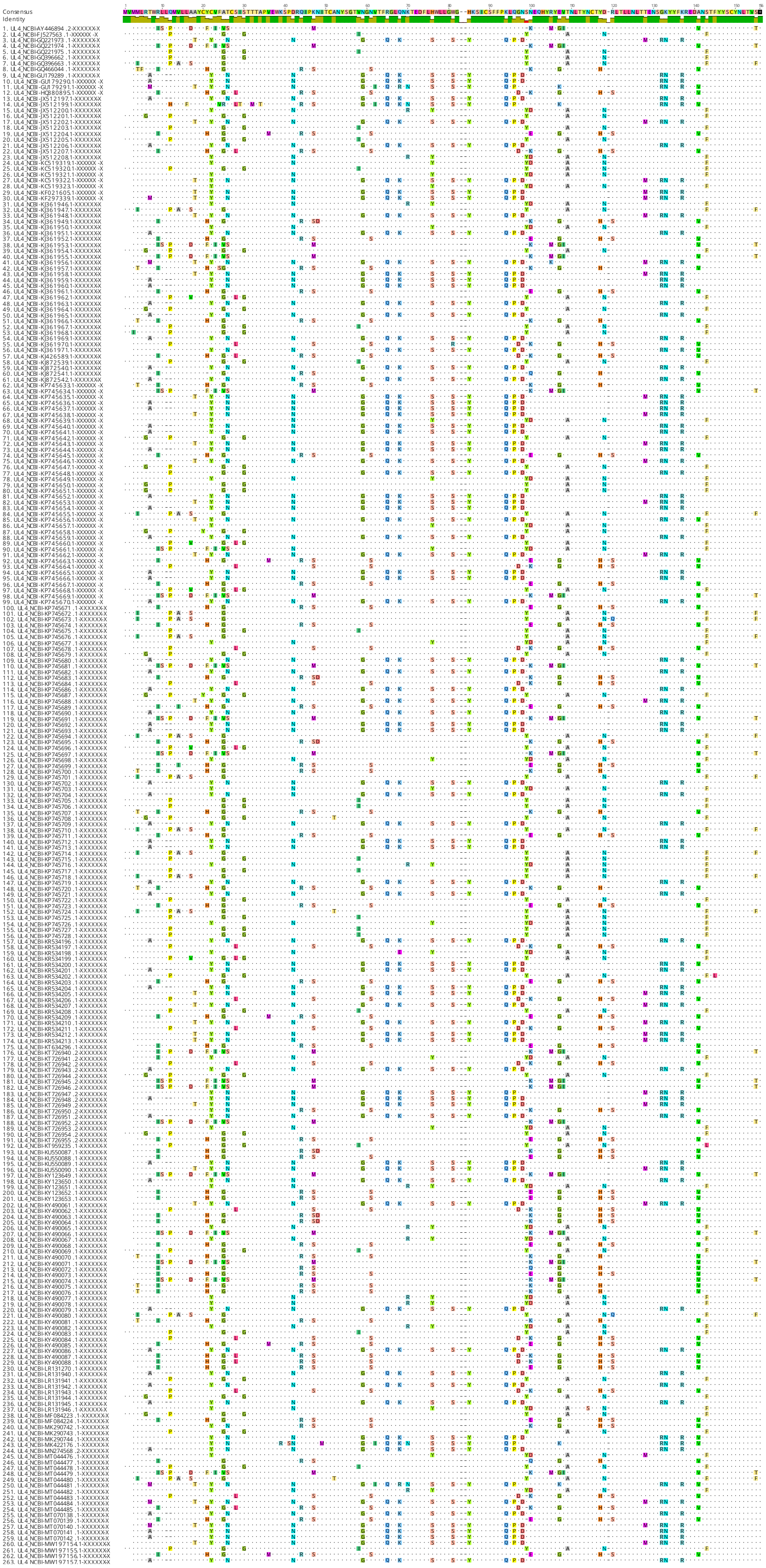

Figure S2. Alignment of all gpUL4 sequences from HCMV genomes in Genbank. Bases differing from the consensus are highlighted, identical residues are shown as '.'

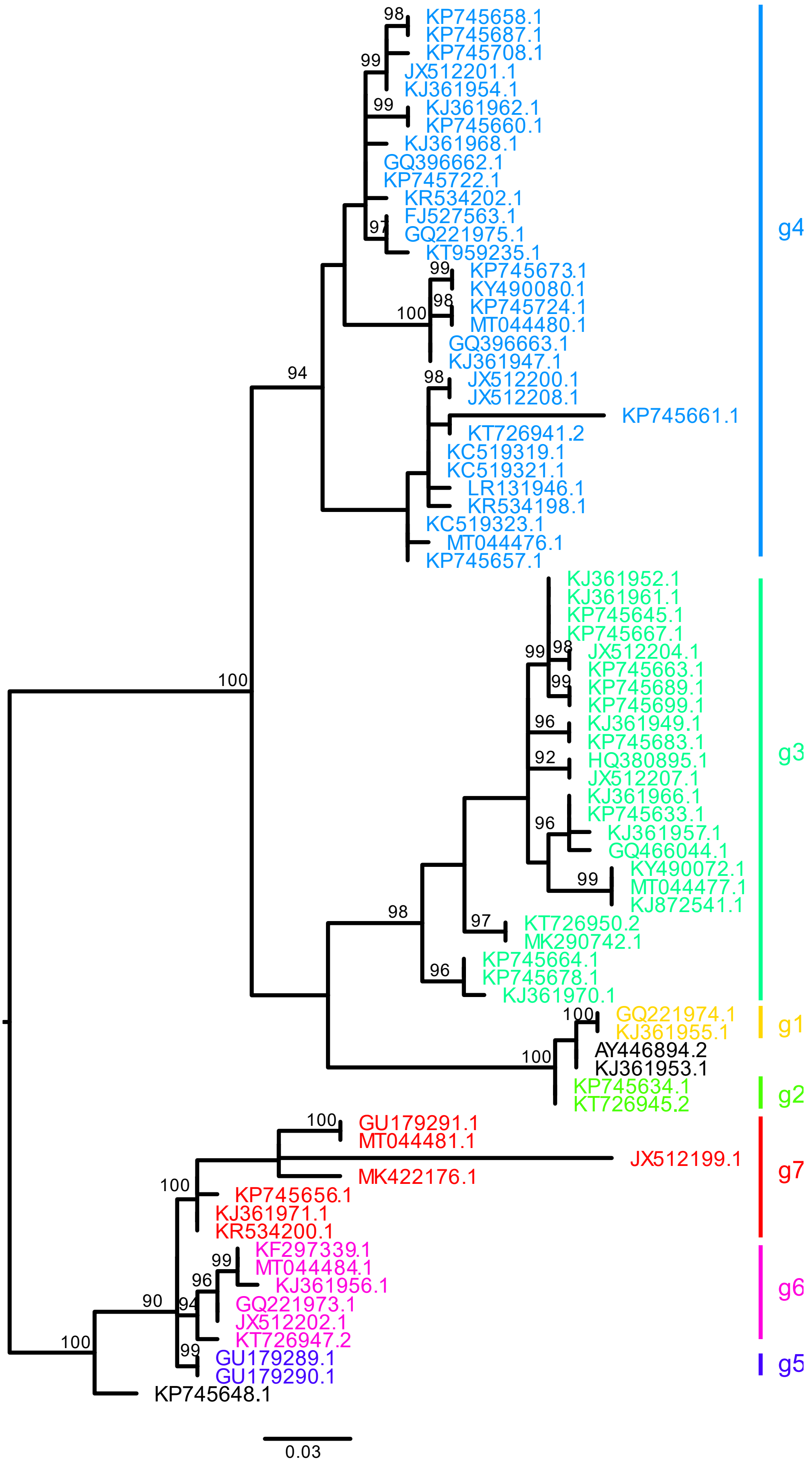

Figure S3. Maximum-likelihood phylogeny of the UL4 proteins. The colours represent the different genotypes defined using ClusterPicker based on a genetic distance threshold of 0.1 and a clade support of 0.9. The genotype numbers are arbitrarily assigned. Bootstrap support above 90 are shown at the nodes. The scale bar for the branch lengths represents the number of amino acid substitutions per site, the tree is midpoint rooted. The majority of UL4 sequences fall within seven well supported phylogenetic clusters (bootstrap support>90) with protein sequences within each cluster having genetic distances below 10%. Two additional clusters, one with a single sequence (KP745648) and another with AY446894 and KJ361953, did not fit within the defined genotypes due to insufficient sequence similarity with currently available sequences.
